# Motion Biomarkers Showing Maximum Contrast Between Healthy Subjects and Parkinson's Disease Patients Treated With Deep Brain Stimulation of the Subthalamic Nucleus. A Pilot Study

**DOI:** 10.3389/fnins.2019.01450

**Published:** 2020-02-07

**Authors:** Andreas Kuhner, Isabella Katharina Wiesmeier, Massimo Cenciarini, Timo Leon Maier, Stefan Kammermeier, Volker Arnd Coenen, Wolfram Burgard, Christoph Maurer

**Affiliations:** ^1^Department of Computer Science, University of Freiburg, Freiburg im Breisgau, Germany; ^2^BrainLinks BrainTools, Cluster of Excellence, University of Freiburg, Freiburg im Breisgau, Germany; ^3^Department of Neurology and Neurophysiology, University Medical Center Freiburg, Medical Faculty, Freiburg im Breisgau, Germany; ^4^Klinikum der Universität München, Ludwig-Maximilians-Universität LMU, Neurologische Klinik und Poliklinik, Munich, Germany; ^5^Department of Stereotactic and Functional Neurosurgery, University Medical Center Freiburg, Medical Faculty, Freiburg im Breisgau, Germany

**Keywords:** Parkinson's disease, machine learning, motion, algorithm, accelerometry

## Abstract

**Background:** Classic motion abnormalities in Parkinson's disease (PD), such as tremor, bradykinesia, or rigidity, are well-covered by standard clinical assessments such as the Unified Parkinson's Disease Rating Scale (UPDRS). However, PD includes motor abnormalities beyond the symptoms and signs as measured by UPDRS, such as the lack of anticipatory adjustments or compromised movement smoothness, which are difficult to assess clinically. Moreover, PD may entail motor abnormalities not yet known. All these abnormalities are quantifiable via motion capture and may serve as biomarkers to diagnose and monitor PD.

**Objective:** In this pilot study, we attempted to identify motion features revealing maximum contrast between healthy subjects and PD patients with deep brain stimulation (DBS) of the nucleus subthalamicus (STN) switched off and on as the first step to develop biomarkers for detecting and monitoring PD patients' motor symptoms.

**Methods:** We performed 3D gait analysis in 7 out of 26 PD patients with DBS switched off and on, and in 25 healthy control subjects. We computed feature values for each stride, related to 22 body segments, four time derivatives, left–right mean vs. difference, and mean vs. variance across stride time. We then ranked the feature values according to their distinguishing power between PD patients and healthy subjects.

**Results:** The foot and lower leg segments proved better in classifying motor anomalies than any other segment. Higher degrees of time derivatives were superior to lower degrees (jerk > acceleration > velocity > displacement). The averaged movements across left and right demonstrated greater distinguishing power than left–right asymmetries. The variability of motion was superior to motion's absolute values.

**Conclusions:** This small pilot study identified the variability of a smoothness measure, i.e., jerk of the foot, as the optimal signal to separate healthy subjects' from PD patients' gait. This biomarker is invisible to clinicians' naked eye and is therefore not included in current motor assessments such as the UPDRS. We therefore recommend that more extensive investigations be conducted to identify the most powerful biomarkers to characterize motor abnormalities in PD. Future studies may challenge the composition of traditional assessments such as the UPDRS.

## Introduction

Technology-based assessments of Parkinson's disease (PD) symptoms can provide valid and accurate parameters of the disease's clinically relevant features (Maetzler et al., 2016). Moreover, they may deliver an additional benefit by detecting, quantifying, and ranking signs and symptoms that have not been considered, or of which we have been unaware before. Motion capture techniques as a typical example for technology-based assessments have already been tested in PD (e.g., Lorincz and Welsh, 2005). One reason for PD being a pioneering disease for motion capture is that PD presents rather clear-cut, familiar motor deficits (Braak et al., 1996; Fahn, 2003; Bloem et al., 2004; Vaugoyeau and Azulay, 2010). They include the classical symptoms such as bradykinesia, rigidity and tremor, freezing, and falling (Bloem et al., 2004; Lewis and Barker, 2009). In addition, PD patients have difficulty in initiating movements and maintaining fluid sequential or repetitive movements. All these motor abnormalities converge to abnormal movement patterns, e.g., during gait. Specifically, PD patients' gait abnormalities consist of decreased gait velocity with shuffling, dragging steps, short step lengths, forward-stooped posture, decreased arm swing, and a wide step variability (Hausdorff et al., 1998; Dietz et al., 2001; Gutnik et al., 2005). Motor deficits usually appear one-sided and remain dominant on one side throughout disease progression (Lewek et al., 2010; Roggendorf et al., 2012; Boonstra et al., 2014, 2016; Plate et al., 2015).

Motion capture techniques were applied in the past to reproduce clinical findings by using either single sensors (accelerometers, inertial sensors, or gyroscopes), e.g., placed on the lower back (Hubble et al., 2015; Bernad-Elazari et al., 2016) or multiple sensors like the Xsens MVN suit used in the present study.

Despite the abundance of available motion data in PD patients, motion capture techniques have not been regularly used in hospitals thus far. One reason for this may be the “big data” problem. Motion data from the Xsens MVN suit used here delivers data on 22 segments, in 6 dimensions (3 rotations, 3 translations), with a frame rate of 120 Hz, so 5 min recording time leads to about 5 million data points. To characterize PD patients' motor abnormalities meaningfully, the amount of data must be considerably reduced, e.g., via feature extraction (Hester et al., 2006; Patel et al., 2009).

Resulting motion features may include parameters such as mean displacements, velocities, and accelerations, or smoothness, represented by jerk (third time derivative of displacement). There is already evidence that jerk is abnormal in PD (Teulings et al., 1997; Hogan and Sternad, 2009). Other methods of data reduction by feature extraction involve signal processing methods, e.g., wavelet analysis (Joshi et al., 2017), stochastic models, like the Hidden Markov Model (Joshi et al., 2017), or machine-learning algorithms (Wouda et al., 2016), i.e., using Random Forests (Wahid et al., 2015; Kuhner et al., 2016, 2017).

Simple machine-learning algorithms like Random Forests may be able to deliver a classification strategy and successfully separate healthy subjects from patients. However, the process by which machine-learning algorithms favor certain features over others is not necessarily instructive. For example, features not applied for a given classification task may either correlate very closely with features already in use (and that were therefore disregarded) or, on the contrary, on features that do not facilitate the classification task at all. As a consequence, machine-learning algorithms are usually unsuitable to advance the understanding of a certain abnormality.

When experimenting with machine-learning algorithms for feature extraction in PD subjects (Kuhner et al., 2016, 2017), we came up with a very simple question that machine-learning classification methods alone cannot answer, namely, which signals or features best describe the difference between healthy subjects on the one hand and PD patients on the other hand.

For this study, we collected gait data of patients with deep brain stimulation (DBS) electrodes in place, switched on or off. For the sake of simplicity, we report here the maximum contrast between healthy subjects, and PD patients with DBS switched on or off. We attempted to optimize a computational model based on a minimally reduced number of ideally one optimal body segment, one single optimal time derivative (displacement, velocity, acceleration, or jerk), and one optimal signal entity (single channels vs. left–right difference) as either absolute values or their variability. In addition, we used AdaBoost to determine the most valuable feature combination to characterize PD patients' state.

## Materials and Methods

### Subjects

This study involved 26 PD patients and 25 healthy control subjects. PD patients stayed in the Department of Neurology and Neurophysiology of the University Hospital Freiburg for their first post-implantation parameter setting of DBS. Among the recorded 26 patients, 7 patients [3 female, 4 male; mean age 58 ± 14.5 years (± SD), age range 40–74 years] with rather long disease duration (see Table 1) were able to complete the 10 m walk test with DBS both in the ON and OFF condition. The remaining 19 patients could not walk 10 m in the OFF condition due to severe postural instability and gait disturbance, and were thus excluded from data analysis.

**Table 1:** Summary of clinical data: Parkinson's disease (PD) patients are shown with type [one patient tremor dominant (TD), six patients akinetic-rigid with dominant postural instability and gait disturbance over tremor (PIGD)], age, disease duration, Unified Parkinson's Disease Rating Scale (UPDRS), with modified subscore III when nucleus subthalamicus (STN) simulation was switched on and off, levodopa equivalent daily dosage (LEDD), most affected side, and of freezing of gait (FOG).

**Patient**	**PD type**	**Age (years)**	**Disease dur. (years)**	**UPDRS deep brain stimulation (DBS) On**	**UPDRS DBS Off**	**LEDD**	**Affected side**	**FOG**
P002	TD	74	9	15	20	300	Left	–
P013	PIGD	69	8	29	38	400	Left	–
P017	PIGD	74	12	22	43	320	Right	+
P018	PIGD	49	9	32	51	250	Left	+
P020	PIGD	57	14	15	47	200	Right and left	–
P021	PIGD	40	10	16	61	350	Right	+
P022	PIGD	43	10	28	45	450	Right	–
Mean		58	10,5	24	44	324		
SD		14.5	2.0	6.5	12	79		

Mean Unified Parkinson's Disease Rating Scale (UPDRS) in the DBS OFF condition was 44 [±11.7 (±SD)], and in the ON condition, 24 [±6.5 (±SD)]. Disease duration ranged from 8 to 14 years [mean 10.5 years ± 1.9 (±SD), Table 1]. The PD patients were measured twice (in the DBS ON and OFF condition) in order to balance on and off DBS conditions. Healthy control subjects [13 female, 12 male; mean age 52 ± 6.8 years (±SD), age range 37–63 years] were recruited from relatives and department staff. All patients and subjects gave their written informed consent in accordance with the Declaration of Helsinki. The study protocol was approved by the Ethics Committee of the University of Freiburg. All the included data were anonymized.

### Experimental Setup

**Figure 1:**
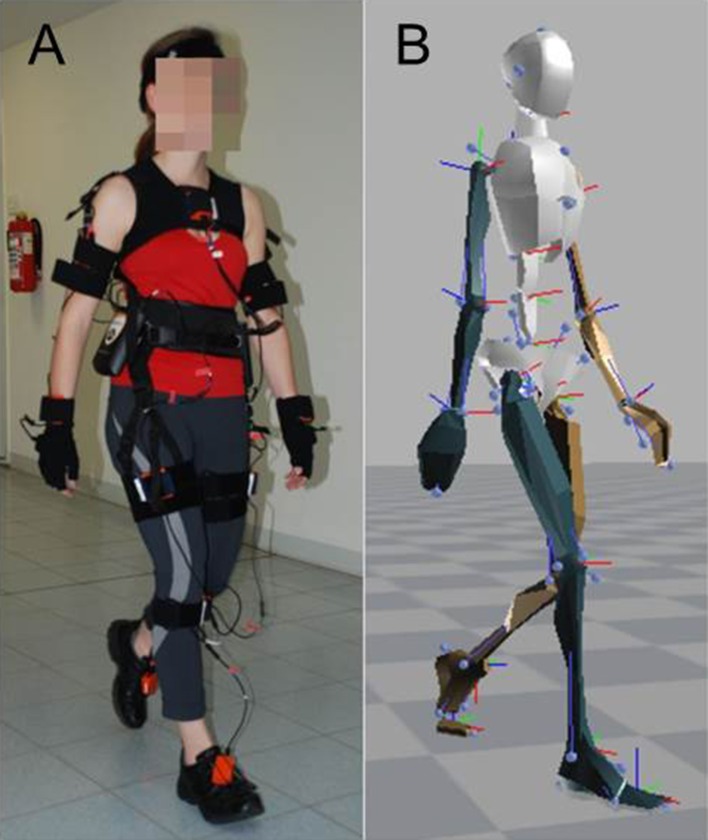
Experimental setup. **(A)** Subject wearing the Xsens sensor suit, consisting of 17 MEMS (microelectro-mechanical systems) which merge the signals of 3D inertial measurement units (IMUs), i.e., linear accelerometers, 3D magnetometers, and 3D rate gyroscopes. Data were sampled at 120 Hz and used to reconstruct 3D segmental movements and joint angles. **(B)** Reconstructed avatar of the subject as a visualization of the segment and joint positions at a given moment.

The Xsens MVN suit is a human motion capture system (see Figure 1) consisting of 17 MEMS (microelectro-mechanical systems) which merge the signals of 3D inertial measurement units (IMUs), i.e., linear accelerometers, 3D magnetometers, and 3D rate gyroscopes. Each MEMS was attached to a specific body region, i.e., the head, upper or lower arms, spine or upper or lower legs, etc. The sensors were positioned next to bigger joints (e.g., knee, wrist, shoulder). Data were sampled at 120 Hz and sent to two wireless receivers. Both receivers delivered the data to a portable computer. Custom-made software employed the data from the sensor trajectories to extrapolate segment size, segment movements, and orientations, as well as joint positions. That data were then used to reconstruct 3D segmental movements and joint angles. Furthermore, the program provided velocity and acceleration for each segment/joint, as well as the orientation. Figure 1 shows a subject's reconstructed avatar as a visualization of the segment and joint positions at a given moment.

### Data Analysis

For data analysis, we applied the absolute values of position, velocity, acceleration, and jerk vectors of segments and joints. The reconstructed skeleton (see right panel in Figure 1) consisted of 22 segments, including 3D positions and orientations in space, which were represented in quaternions. The data sets covering gait were split into individual steps and strides (*j* ∈ *S*). We first identified the exact moment when the swinging leg passes the standing leg. Starting from that point in time, we went both forwards and backwards in time until we identified the moments where the foot segment's absolute velocity dropped below 5% of its maximum velocity, which then identified the start and end of a given step. Each stride served as an individual data set for further processing.

#### Data Preprocessing

We denoted with $p_{i}^{\left(t\right)}$the position of segment *i* at time step *t*. A Gaussian filter with several time dependent positions was used to filter sensor-related noise to compute a smoothed position trace ${\underline{p}}_{i}^{\left(t\right)}=\frac{\left(\sum_{n=-\tau }^{\tau }\frac{1}{\sqrt{2\cdot \pi \cdot \sigma^{2}}}\cdot e^{-\frac{1}{2}\cdot \frac{n^{2}}{\sigma^{2}}}\cdot p_{i}^{\left(t+n\right)}\right)}{\left(\sum_{n=-\tau }^{\tau }\;\frac{1}{\sqrt{2\cdot \pi \cdot \sigma^{2}}}\cdot e^{-\frac{1}{2}\cdot \frac{n^{2}}{\sigma^{2}}}\right)},$ where the index *i* denotes the segment ID. In the next step, we transformed position traces into the local frame of the hip, namely ${\hat{p}}_{i}^{\left(t\right)}={\underline{p}}_{i}^{\left(t\right)}-{\underline{p}}_{0}^{\left(t\right)}$, where ${\underline{p}}_{0}^{\left(t\right)}$ is the smoothed position of the hip frame. This transformation allowed us to evaluate data such as the arm swing in relation to the lower trunk to guarantee independence from the main movement direction.

#### Features

The 22 segments consisted of 6 midline segments (trunk) and 8 segments with left and right specificity in each case, e.g., left and right hand, thus 16 segments with left and right specificity in total. We can reformulate the segment positions as $\left\{{\hat{p}}_{1}^{\left(t\right)},\ldots ,{\hat{p}}_{6}^{\left(t\right)},{\hat{p}}_{7,l}^{\left(t\right)},{\hat{p}}_{7,r}^{\left(t\right)},\ldots ,{\hat{p}}_{14,l}^{\left(t\right)},{\hat{p}}_{14,r}^{\left(t\right)}\;\right\}.$

The first group of features includes the means between the left and right side of the segments with left and right specificity, i.e.,

$$\begin{array}{l} f_{i,m}^{\left(t\right)}=\frac{1}{2}\cdot \left({\hat{p}}_{i,l}^{\left(t\right)}+{\hat{p}}_{i,r}^{\left(t\right)}\right)\text{for}\;i\in \left\{7,\ldots ,14\;\right\}. \end{array}$$

The second group of features covers the left/right differences, i.e., $f_{i,d}^{\left(t\right)}=\left({\hat{p}}_{i,l}^{\left(t\right)}-{\hat{p}}_{i,r}^{\left(t\right)}\right)$ for *i* ∈ {7, …, 14}. Using the second feature, we evaluated differences between the more and less affected side.

Furthermore, we computed the first and second moment of each feature for each body segment *i* over the whole trajectory, yielding each feature's mean and variance. The mean is computed by $\mu_{i}=\frac{1}{T}\cdot \overset{T}{\underset{t\;=\;1}{\sum }}d_{i}^{\left(t\right)}$ for *i* ∈ {1, …, 22} and the variance with $\sigma_{i}^{2}=\frac{1}{T-1}\cdot \overset{T}{\underset{t\;=\;1}{\sum }}{\left(\mu_{i}-d_{i}^{\left(t\right)}\right)}^{2}$ for *i* ∈ {1, …, 22} where *d* is either *p, f*_*i*_ or *f*_*m*_.

Thus, the total number of features is the product of the absolute mean and variance (2) of a single stride, times body segments (22), times displacement and three orders of time derivative of displacement (4), times left–right mean and left–right difference (2) of 8 out of 22 segments, resulting in 264 features. One subject's data set consisted of values for all 264 features for each stride. Since we evaluated about 30 strides for each subject, the total amount of data points of one subject amounted to a value of about 30 × 264 = 7,920.

#### Weak Classifiers

A weak classifier (*C*_*W*_) relates to a separating algorithm which splits a certain feature of the data into two categories with an accuracy of at least 50% (e.g., a specific segment's slow vs. fast velocity). Parameters {−1, 1} are chosen as class labels. Weak classifiers were used to detect a specific threshold that delivered the best separation results between PD patients and healthy control subjects. We calculated the highest classification rate by uniting data sets of healthy subjects and PD patients into one, sorting the elements according to the value of the current separating algorithm, and taking the value ε as a threshold between two elements in the set. The final weak classifier is calculated by *WC*_*i*_(*x*) = 1 *if x* < ε_*i*_ *and WC*_*i*_(*x*) = −1 *ifx* ≥ ε_*i*_.

The classifiers' performance was quantified by a leave-one-subject-out cross-validation, i.e., one subject was removed from the data set, and the residual data set was used to predict the missing data sample. This procedure was repeated for each subject.

#### Meta-Classifier AdaBoost

We used AdaBoost to evaluate which types of feature combinations ameliorated the classification results as compared to single features. AdaBoost works as a meta-classifier and combines multiple classifiers, i.e., features plus respective thresholds, into one, by weighting the output of this set of weak classifiers. In this case, the meta-classifier represents an optimal mix of features for maximum differences between PD patients and healthy subjects. If the result is smaller than zero, the data set belongs to the first group (here, PD patients); otherwise it belongs to the second group (healthy subjects). In principle, AdaBoost repeats the following two steps: First, the algorithm identifies a classifier *C*_*m*_ which contributes the most information to the current weighted sum of chosen weak classifiers $C^{\left(m-1\right)}=\overset{m-1}{\underset{k=1}{\sum }}\alpha_{k}C_{k},$ i.e., $C_{m}=\underset{c\in C_{W}}{argmin}\;\underset{c\left(x_{i}\right)\;\neq \;y_{i}}{\sum }\omega_{i}^{\left(m\right)}$ where $\omega_{i}^{\left(1\right)}:=1,\omega_{i}^{\left(m\right)}:=e^{-y_{i}\cdot C^{\left(m-1\right)}\left(x_{i}\right)}$ for *m* > 1. Then, the sum runs over the total training set. Thereby, ${\left\{\left(x_{i},y_{i}\right)\right\}}_{i=1}^{N}$ denotes the set of training data points *x*_*i*_ and the corresponding labels *y*_*i*_. The concept of this algorithm is to add the classifier which maximizes information acquisition. In this way, the algorithm categorizes the wrongly classified elements of *C*^(*m*−1)^ correctly. The second step of the AdaBoost involved the computation of a weight for the selected classifier. Let $\xi^{\left(m\right)}=\overset{N}{\underset{i\;=\;1}{\sum }}\omega_{i}^{\left(m\right)}$ be the total error. Note that the optimal weight α_*m*_ for the classifier *C*_*m*_ is given by $\alpha_{m}=\frac{1}{2}\cdot log\left(\frac{1-\epsilon_{m}}{\epsilon_{m}}\right)$ with $\epsilon_{m}=\frac{\underset{C_{m}\left(x_{i}\right)\neq y_{i}}{\sum }\omega_{i}^{\left(m\right)}}{\xi^{\left(m\right)}}$. The resulting function to classify a sample *x* is: $C^{\left(m\right)}\left(x\right)\;=\;\overset{m}{\underset{k=1}{\sum }}\alpha_{k}\cdot C_{k}\left(x\;\right).$ The algorithm chooses the most accurate weak classifier as first classifier. Each succeeding classifier is not necessarily a classifier with high accuracy, but it does add the highest amount of information to the existing (chosen) set. As a result, a set of weak classifiers represents one of the best combinations for separating the data.

Our classifier accuracy values were statistically analyzed using the JMP® statistic program by SAS Institute Inc., Cary, NC, USA. We tested normal distribution and homogeneity of variances with the Kolmogorov–Smirnov test and parametric methods for further analyses. Due to the expected dependency between the outcome measures within motor behavior, statistical significance was tested by an analysis of variance (ANOVA). The within-subjects factors were: (i) absolute values vs. variance of absolute values, (ii) displacement vs. velocity vs. acceleration vs. jerk-related measures, (iii) mean absolute values vs. left–right difference, and (iv) segments (e.g., head, neck, shoulder). The level of statistical significance was set at *p* = 0.05. Differences between groups were tested using Tukey's *post-hoc* test, if multiple comparisons were considered.

## Results

**Figure 2:**
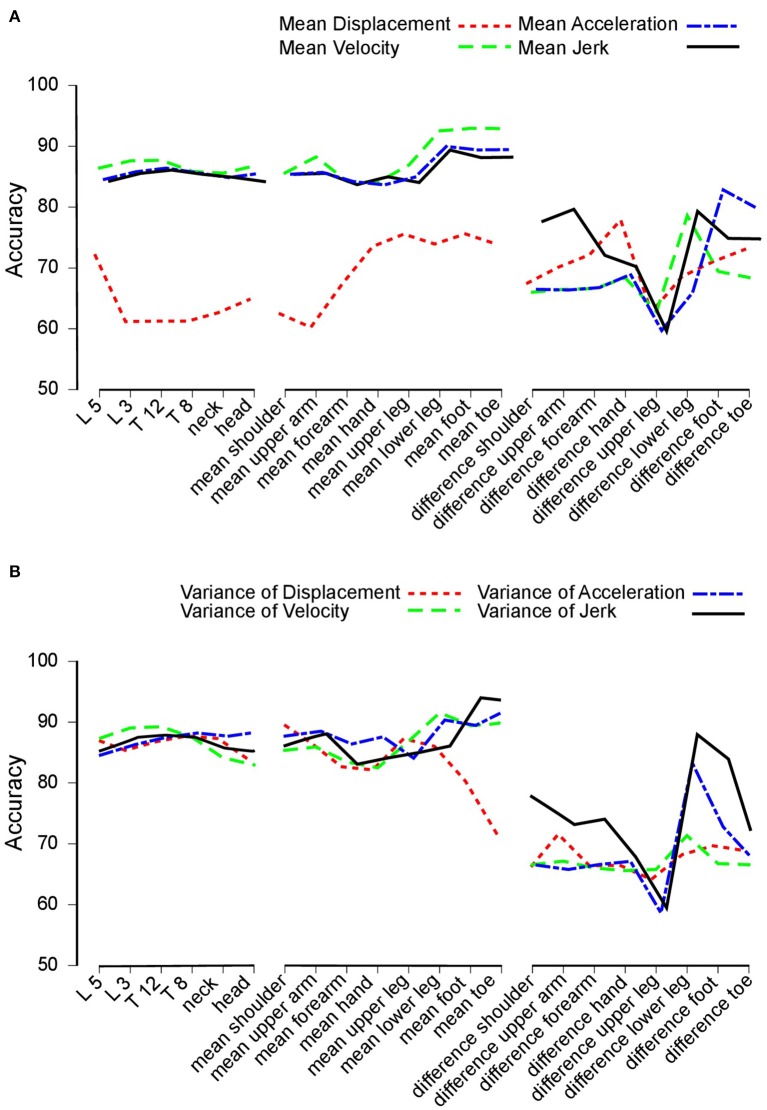
Accuracy. **(A)** Accuracy achieved to distinguish between Parkinson's disease (PD) patients and healthy control subjects. Accuracy for trunk segments (left section), limb segments (central section), and limb segment asymmetries (right section) using mean displacement (dotted line), velocity (dashed line), acceleration (dashed and dotted line), and jerk (solid line). **(B)** Accuracy values based on variance measures. This figure is based on variance measures instead of mean values for displacement (dotted line), velocity (dashed line), acceleration (dashed and dotted line), and jerk (solid line).

Overall, our study yielded a total of four different time derivatives: displacement, velocity, acceleration, and jerk (Figure 2). Hereby, displacement relied on the position vectors between the hip and the analyzed segment. Velocity, acceleration and jerk were the first, second, and third derivation of that position. Figure 2A depicts the accuracy, which we defined as the percentage of correctly classified subjects as PD patients: Here, nearly all features [except for the displacement of the midline segments (trunk) and the mean between left and right limbs] attained accuracy rates between 80 and 90%.

Figure 2B displays the results of the variance of features. Again, features of the midline segments and the mean between more and less affected limb performed across all time derivatives equally with accuracy rates of 80% to 90%.

### Systematic Evaluation of Feature Characteristics

**Figure 3:**
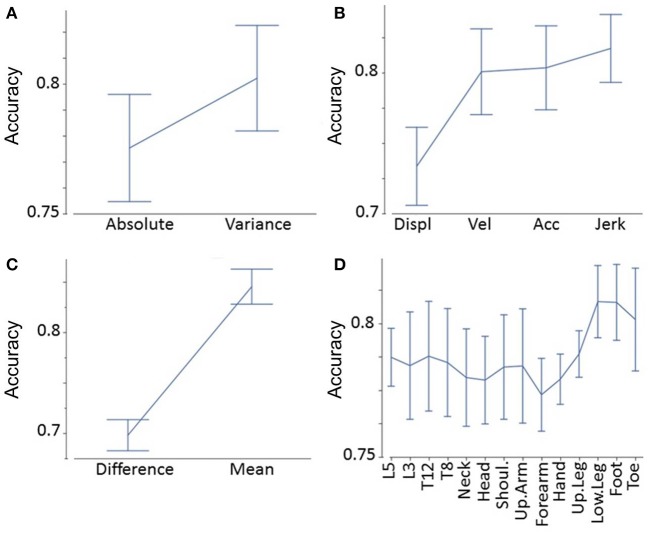
Accuracy analyses. Accuracy as a function of absolute values vs. variance of absolute values **(A)**, displacement vs. velocity vs. acceleration vs. jerk-related measures **(B)**, mean absolute values vs. left–right difference **(C)**, and segments **(D)**.

We applied accuracy values to determine the effects of different degrees of freedom in our feature matrix, i.e.,:

- Absolute values vs. variance of absolute values (Figure 3A)
- Displacement vs. velocity vs. acceleration vs. jerk-related measures (Figure 3B)
- Mean absolute values vs. left–right difference (Figure 3C)
- Segments (e.g., head, neck, shoulder, Figure 3D).

We found that variance of a certain feature determined significantly higher accuracy values than the raw feature (*F* = 26, *p* < 0.0001, Figure 3A). Jerk measures displayed significantly higher accuracy values than acceleration, velocity, and displacement-related measures (*F* = 32, *p* < 0.0001, Figure 3B). *Post-hoc* tests showed that displacement-related measures reveal significantly less accuracy than all other time derivatives (*p* < 0.0001 between displacement, and all other time derivatives). The limbs' mean values demonstrated significantly higher accuracy values than the differences between the more and less affected limb (*F* = 210, *p* < 0.0001, Figure 3C). Finally, the accuracy of segments varied: lower leg, foot, and toe measures revealed the greatest accuracy, followed by trunk measures (L5, L3, T12, T8); the upper arm, shoulders, head, hand, and forearm were lowest (*F* = 2.3, *p* = 0.01, Figure 3D). *Post-hoc* tests revealed that both the foot and lower leg showed significantly higher accuracies than the forearm (*p* = 0.043 and *p* = 0.045, respectively). All other pairwise comparisons between segments did not reach a significance level below *p* = 0.05. These findings were in line with the overall highest accuracy value (variance of jerk of the mean of the left and right foot).

### Meta-Classifier AdaBoost

**Figure 4:**
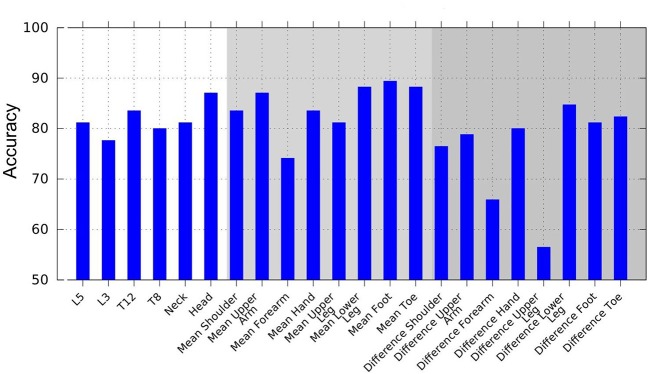
Accuracy achieved using AdaBoost, based on combinations of multiple weak classifiers to create a stronger classification hypothesis. Weak classifiers are displacement, velocity, acceleration, and jerk mean, and variance features.

When evaluating the performance of combinations of classifiers using AdaBoost, we observed the following rules: The feet yielded the highest accuracy by applying a combination of variance of jerk, variance of acceleration, and mean of velocity. Concerning the left–right difference, the combination of variance of jerk and the mean of displacement displayed the highest accuracy. The segment with the overall highest accuracy was the lower leg using the combination of variance of acceleration, jerk, and mean of velocity. Of the head and trunk segments, the head yielded the highest accuracy rate based on the combination of variance and mean of acceleration and displacement. Overall, the head and trunk segments' best feature combination was mean of displacement, variance of jerk, and acceleration. As an example of feature combinations, Figure 4 illustrates the combined AdaBoost accuracy rates separated by segments. Note that due to the cross-validation procedure, the overall performance of AdaBoost is lower than the best feature of each segment.

## Discussion

Gait deficits are one of the major problems that determine PD patients' quality of life. PD patients report them as the most debilitating disease features (Horak et al., 2005). While gait disturbances occur in all PD subtypes, they are the leading symptom in the postural instability and gait disturbance type of PD, PIGD. The patient group studied here mainly presented akinetic types of the disease. In this paper, we analyzed gait because it involves movements of all body segments. Other motion tasks, e.g., getting up from a chair or doing accuracy tasks with the hand, require specific instructions. In addition, they represent a minor portion of overall motion deficits and vary widely.

The aim of this pilot study was to compare features from sensor suit data (segments, time derivatives, variance, left–right difference) that enable optimal discrimination between the gait patterns of advanced-stage PD patients with DBS electrodes in the nucleus subthalamicus (STN) switched on or off, and healthy subjects. Other approaches to classify PD patients' gait employed data from considerably different sources for different purposes. While Khorasani and Daliri (2014) and Joshi et al. (2017) analyzed data of force-sensitive insoles provided by Hausdorff et al. (1998), Wahid et al. (2015) used whole body gait data and force platform outcomes to optimize classification results. Here, we report the maximum contrast between healthy subjects and PD patients with DBS switched on or off. In theory, one could calculate the contrast between many different subgroups of this cohort. For example, another potential question is which parameter best separates PD patients with DBS switched off from those with DBS switched on. Such a contrast would provide information on the treatment effect of DBS which might deliver another favorable signal. Here, we focused on the aforementioned contrast for simplicity's sake.

In our approach, we calculated weak classifiers for each feature separately in order to compare the quality of different features. In the next step, we systematically analyzed the accuracy of those classifiers to correctly distinguish PD patients from healthy subjects. In general, we identified an obvious grade of classifier accuracies: (i) variance was superior to absolute values of body motion, (ii) jerk (third time derivative of displacement) was superior to acceleration, velocity, and displacement, (iii) average motion across left and right was superior to the differences, i.e., asymmetries between left and right markers, and (iv) feet and lower leg segments were superior to trunk, head, and hand movements. These principles were in line with the overall highest accuracy value (variance of jerk of the mean of the left and right foot).

In addition, we evaluated the accuracy outcomes from AdaBoost and the respective sets of weak classifiers to extract the combination of features displaying maximum discriminative power. The main purpose of this approach was to potentially enhance the quality of discrimination by combining different features across different segments.

Meta-classifiers like AdaBoost deliver a set of classifiers that optimally separate the group of PD patients from healthy subjects. We found that a combination of two to three features is optimal. In most cases, the combination consisted of a feature related to absolute values with a low order of time derivative, like displacement or velocity, and a variance-related feature of a high order of time derivative like acceleration or jerk. This proved to be true for the trunk and limb, as well as for the asymmetry features. Out of all head and trunk segments, the head delivered the highest accuracy rates.

In a previous study, we showed that machine-learning approaches significantly correlate with known clinical measures such as the UPDRS (Kuhner et al., 2017).

Our selection of a small set of features instead of using the entire body data set is specifically interesting since a full sensor suit is obtrusive for everyday use. On the other hand, very few sensors (e.g., at the belt, or as a collar, at the wrist, near the trunk) to monitor motion patterns in PD patients (Horak et al., 2005) and other diseases (Bonora et al., 2015) might neglect important information of certain motor tasks, particularly considering the growth in wearable technology in conjunction with modern smartphones. Our findings may facilitate the development of motion capture systems based on commercial-grade wearable sensors and “smartphone apps” to observe motor features in PD patients. In addition, data reduction is often done locally, which means next to the sensor (IMU), before the information is transferred to a collecting unit and further processed, due to the usually limited rate of data transfer. Such data reduction means the signals of interest must be pre-selected. We aimed here to make recommendations as to where best to place sensors and which type of signals should ideally be processed so as to exploit the available motion data to the maximum.

### Limitations

In this study, PD patients were suffering from advanced-stage PD and were undergoing recent DBS of the STN. Most study participants were not able to walk 10 m in the OFF condition independently, which greatly reduced our sample size and potentially biased our data. This severely affected group was chosen to analyze the strongest expressions of pathological features. Early-stage PD gait deficits, PD patients without DBS, and the variability of the defined features during medication were not the focus of the present study. Additional investigations should explore whether our study's optimal-parameter findings also apply to less severely affected PD patients and other treatment conditions.

## Conclusions

Our approach proposes a specific marker position (foot, lower leg) and certain data processing algorithms (variance of jerk) to optimally characterize PD patients' motion abnormalities during walking. Using AdaBoost, we identified sets of classifiers that optimally separate PD from healthy subjects. For walking, a useful combination of classifiers may refer to the head and a foot segment. Moreover, this combination should include an absolute value derived from a low order of time derivative and a variance-related feature from a high order of time derivative.

In the future, we aim to evaluate the differential effect of treatment (e.g., STN DBS) in order to characterize the optimal set of features for monitoring intervention effects, before we extend this approach to different motion patterns, e.g., standing up from a chair, turning around, and interacting with the surroundings. In future studies, our results may help to develop a low-threshold and objective analysis tool for diagnosing and monitoring motor abnormalities in PD. Given our latest findings, a simplified and small sensor attachable to the shoe may suffice to analyze PD gait abnormalities.

More generally, this small pilot study ranked motion features according to their distinguishing power and identified the variability of a smoothness measure i.e., jerk of the foot as the most favorable signal from which to separate healthy subjects' from PD patients' gait. This biomarker is imperceptible to clinicians' naked eye and, therefore, is not incorporated in current motor assessments such as the UPDRS. Consequently, we believe that more extensive investigations are warranted to identify the most powerful biomarkers for characterizing motor abnormalities in PD. Future studies may ultimately challenge how traditional assessments such as the UPDRS are composed.

## Data Availability Statement

The datasets generated for this study are available on request to the corresponding author.

## Ethics Statement

All patients and subjects gave their written informed consent in accordance with the Declaration of Helsinki. This study protocol was approved by the Ethics Committee of the University of Freiburg. All data was included anonymized.

## Author Contributions

AK and CM were responsible for the concept and design of the work, the analysis and interpretation of data, and the first draft of the manuscript. AK and TM were responsible for data acquisition. MC contributed to conceiving and designing the work, and critically revised the manuscript. IW drafted the manuscript, edited the final manuscript for submission, and revised the work critically. SK, VC, and WB contributed to the concept and critical revision of the work. All authors approved the final manuscript.

### Conflict of Interest

The authors declare that the research was conducted in the absence of any commercial or financial relationships that could be construed as a potential conflict of interest.
